# Fear of Cancer Recurrence and Fear of Cancer Progression, Digital Resource Engagement and Health Literacy: A Review

**DOI:** 10.3390/curroncol31120559

**Published:** 2024-11-29

**Authors:** Maebh Kenny-Jones, Paul Nankivell, Hisham Mehanna, Gozde Ozakinci

**Affiliations:** 1 Division of Psychology, Faculty of Natural Sciences, University of Stirling, Stirling FK9 4LA, UK; maebh.kenny-jones@stir.ac.uk; 2 Institute of Head and Neck Studies and Education, Department of Cancer and Genomic Sciences, University of Birmingham, Birmingham B15 2TT, UKh.mehanna@bham.ac.uk (H.M.)

**Keywords:** fear of cancer recurrence, fear of cancer progression, health literacy, digital resources, engagement, digital health, cancer care

## Abstract

Cancer care is evolving, and digital resources are being introduced to support cancer patients throughout the cancer journey. Logistical concerns, such as health literacy and the emotional experience of cancer, need to be considered. Fear of cancer recurrence (FCR) and fear of cancer progression (FOP) are relevant emotional constructs that should be investigated. This scoping review explored two main objectives: first, the link between FCR/FOP and engagement with digital resources, and second, the link between FCR/FOP and health literacy. A database search was conducted separately for each objective. Relevant papers were identified, data were extracted, and a quality assessment was conducted. Objective 1 identified two relevant papers that suggested that higher levels of FCR were correlated with lower levels of engagement with digital resources. Objective 2 identified eight relevant papers that indicated that higher FCR/FOP is correlated with lower health literacy. However, one paper with a greater sample size and a more representative sample reported no significant relationship. There may be important relationships between the constructs of FCR/FOP, resource engagement, and health literacy and relationships may differ across cancer type and sex. However, research is limited. No studies examined the relationship between FOP and engagement or FCR/FOP and digital health literacy, and the number of studies identified was too limited to come to a firm conclusion. Further research is needed to understand the significance and relevance of these relationships.

## 1. Introduction

Modern cancer care is evolving rapidly. Earlier detection of cancer and improving treatment outcomes have resulted in increased survival with greater long-term healthcare needs [1]. The increased need for access to healthcare places a significant and increasing burden upon current healthcare systems [1]. Alternative solutions are required that reflect increasingly limited resources and encourage a new health model that moves away from a paternalistic system and instead helps patients take an active role in managing their long-term health [2]. This notion has led to an increase in digital health technologies and resources that are made available to individuals. Mobile-based, low-cost resources have been proposed as a crucial tool in lessening health spending and encouraging patients to take a more active role in their care [3]. The scope of these digital resources also ranges massively and can target different parts of the cancer care continuum, from prevention through treatment, symptom management, and survivorship [4].

However, for digital resources to be effective in supporting self-management, the barriers and facilitators to using them must be explored and understood from a patient perspective. The concerns that cancer patients report can include a lack of empowerment and support to use resources, digital incompatibility with their own technology, dislike of content, increased patient burden, difficulties using digital technology, low perceived usefulness, and the inability of digital interventions to replace interpersonal rapport [5,6]. Facilitators include cancer-specific information and communication with health care professionals, contact with fellow patients, symptom monitoring, real-time feedback, tailored information for personal goals, higher perceived usefulness, high usability, and age-appropriate design [5,6,7].

Additionally, an important consideration surrounding digital resources is digital health literacy. This combines two important concepts: health literacy and digital literacy. Health literacy refers to individuals possessing a level of knowledge, understanding, confidence, and the appropriate skills to access health information and services and, in turn, understand, evaluate, and use these services effectively [8]. Digital literacy can be outlined as the ability to access, manage, understand, and communicate information through digital technologies, as well as be able to evaluate this information safely and appropriately [9]. Together, these constructs describe the skills needed for an individual to effectively use digital health resources.

However, levels of health literacy and digital literacy differ among the population. In a cross-sectional survey measuring health literacy among British adults, 19.4% of participants expressed difficulty with written health information, and 23.2% faced challenges discussing health concerns with care providers [10]. For cancer patients, lower health literacy is associated with consequences such as an increased number of hospitalizations, greater emergency care requirements, increased uptake of preventative services, and limited understanding of health information and how to take medication properly [11]. Additionally, according to the 2023 Lloyds Consumer Digital Index Report [12], 25% of the UK population has the lowest digital capability and is likely to struggle when interacting with online services. Additionally, 2.1 million people are offline, and around 4.7 million cannot connect to Wi-Fi. Those with the lowest level of digital literacy are more likely to be over 70, express a lack of interest in online resources, are concerned about online fraud, and do not possess the necessary skills to limit their risk.

However, while these concerns focus on some of the logistical problems of implementing digital resources for cancer patients, it is essential to investigate the influence of the emotional experience of cancer on the use of digital resources as the approach to cancer care continues to evolve. When exploring the vast psychosocial consequences of cancer (e.g., sexual dysfunction [13], impact on employment status [14]), one issue of importance is fear of cancer recurrence (FCR) and fear of cancer progression (FOP). Although labeled separately, FCR and FOP are both concerned with patients’ fears around cancer either coming back, progressing, or metastasizing and share comparable defining features [15]. Therefore, for this review, FCR and FOP will be defined as ‘fear, worry, or concern relating to the possibility that cancer will come back or progress’ [16]. A recent meta-analysis examining the prevalence of FCR in cancer survivors and patients shows that 59% of cancer survivors and patients experience at least a moderate level of FCR, while 19% report a high level of FCR [17]. As rates of cancer survivorship increase, patients live longer while coping with their fears and uncertainties about cancer returning or progressing, making FCR/FOP a critical support need [18].

FCR/FOP can manifest in different ways depending on the individual. At some levels, these fears can be adaptive and encourage patient engagement with treatment, follow-up, and making healthy lifestyle changes [17]. However, excess FCR/FOP is associated with increased and excessive care seeking, hypervigilance around symptoms, or even withdrawal from healthcare, avoidance of appointments, and ignoring questionable symptoms [19]. Clinical levels of FCR can also limit patients’ quality of life and daily functioning [17]. FCR/FOP is a complicated and distressing experience for patients, and the nature of its manifestation means that it is likely to have a direct impact on the uptake of digital cancer resources—whether this is contributing to FCR/FOP through increased symptom checking and overuse of resources or the complete avoidance of resources as a potential trigger for heightened FCR/FOP.

FCR/FOP has been investigated to identify its potential risk factors and predictors. Studies have suggested that younger age, low mood, psychological issues (including anxiety and depression), lower levels of optimism, lower self-esteem, and denial and avoidance-oriented coping can act as predictors and risk factors for FCR [20]. These are important psychological considerations that may well influence patients’ uptake and engagement with digital resources. Furthermore, lower satisfaction in terms of understanding information, symptom management, and care co-ordination are also significant predictors of FCR [17]. Interestingly, these predictors appear to share some similarities with respect to health literacy and may be particularly important when encouraging patients to take an active role in their care using digital resources.

This scoping review has two main objectives. First, we aim to explore the relationship between FCR/FOP and uptake and engagement with digital resources, and second, the relationship between FCR/FOP, health literacy, and digital health literacy.

## 2. Materials and Methods

A scoping review method was used to explore the aims outlined above. This approach was chosen as it allowed for the exploration of study areas that do not appear to have been widely discussed or researched in the literature to date. A scoping review allowed for a general overview of what kind of research has been carried out so far, what some of the initial results indicate, the gaps in research that remain, and whether there is justification to continue research in these specific areas. The scoping review has not been registered.

### 2.1. Objective 1—What Is the Relationship Between FCR/FOP and Engagement with Digital Resources?

#### 2.1.1. Eligibility Criteria

##### Studies

For this scoping review, any studies published in a peer-reviewed journal were considered. Study design was not specified to ensure that any relevant studies that discuss the relationship between FCR/FOP and engagement with digital resources can be considered. The type of digital resource was not specified in the search protocol to open up the search to any study that may be relevant. However, it should be considered that most digital resources produced and then tested on a patient population are interventions aimed at improving some aspect of cancer patients’ lives (e.g., quality of life and mental well-being) [4].

##### Participants

Participants needed to be adults (over 18 years old) who have received a cancer diagnosis. Studies were included regardless of their own participant criteria, including cancer type, cancer stage, or participant demographics.

##### Outcome Measures

Any studies that reported a quantitative assessment of FCR or FOP levels and a measure of engagement with digital resources were considered for initial screening. In this case, the term engagement is used as an umbrella term to quantify the interaction with digital resources. Any outcomes that attempted to measure engagement with digital resources were considered. Measuring engagement is a multidimensional concept and may involve measures such as the number of logins, time spent logged in, time spent on different pages, pages viewed, meeting a minimum threshold of page views, etc. [21]. Studies were included in this review if they expressed that a specific outcome measured was engagement, regardless of how they specifically calculated this. Studies must then provide an analysis of the relationship between FCR/FOP levels and engagement with resources. This had to be clearly reported in the results section as the result of a quantitative analysis.

For this scoping review, we deemed it irrelevant whether FCR/FOP and engagement outcomes were primary or secondary as long as the relationship was reported.

#### 2.1.2. Search Strategy

PubMed, Cochrane, and CINAHL were used as the primary databases for this search. The CINAHL integrated search was used and included APA PsycArticles, APA PsycINFO, Health Source: Nursing/Academic Edition, and MEDLINE. Advanced search was used to input keywords and search titles and/or abstracts. No publication date was specified to ensure any relevant studies were identified. See Table 1 for Objective 1 search terms:

### 2.2. Objective 2: What Is the Relationship Between FCR/FOP and Health Literacy and Digital Health Literacy?

#### 2.2.1. Eligibility Criteria

##### Studies

Once again, any studies published in a peer-reviewed journal were eligible for this review, and studies were not selected based on study design.

##### Participants

As outlined above for Objective 1.

##### Outcome Measures

Studies were considered if they reported a quantitative assessment of FCR/FOP and health literacy or digital health literacy. It was then essential that the study calculated and reported the relationship between FCR/FOP and health literacy or digital health literacy. Studies that reported both measures but did not calculate and report a relationship were excluded.

#### 2.2.2. Search Strategy

PubMed, Cochrane, and CINAHL were the primary databases used. Once again, CINAHL searched APA PsycArticles, APA PsycINFO, Health Source: Nursing/Academic Edition, and MEDLINE. No publication dates specified as focusing on both health literacy and digital health literacy opened up the search to both previous and contemporary studies focusing on these constructs. See Table 2 for search terms for Objective 2:

All papers retrieved by the search across the three databases were exported to a citation manager for screening. Duplicates were removed, and titles and abstracts were screened by one author (MK-J). Once this was completed, full text screening took place to identify relevant articles for the review that met inclusion criteria. In the case of Meng et al.’s [22] study, which was in the German language in the original article, we used an online translation tool and followed it with a German psychologist academic checking the translation for accuracy.

### 2.3. Quality Assessment

All studies included in the review were quality assessed using checklists from the Joanna Briggs Institute, depending on the study design. The checklists used were for analytical cross-sectional studies [23], cohort studies [24], and randomized controlled trials [25]. The checklists were of different lengths, and, therefore, studies were given a number based on the relevant aspects of the checklist. Quality assessments were reported in the data extraction tables, see Section 3.

## 3. Results

As this scoping review aimed to answer two separate research questions, the results of each search will be discussed and reported separately below.

### 3.1. What Is the Relationship Between FCR/FOP and Engagement with Digital Resources?

**Figure 1:**
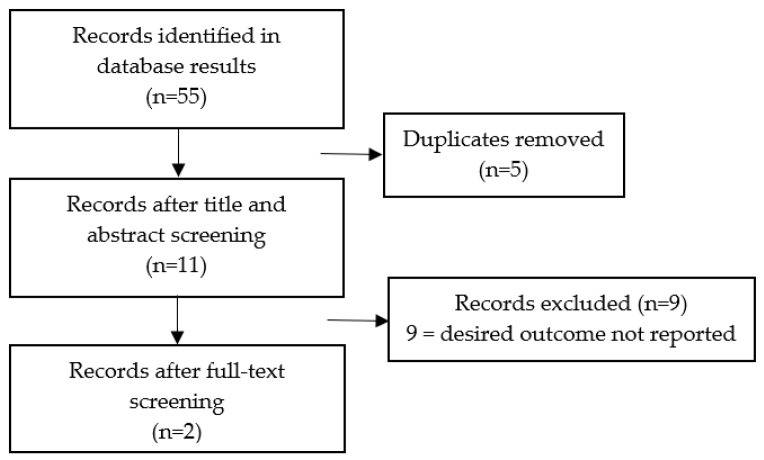
PRISMA flowchart for Objective 1.

Figure 1 displays the screening process and outlines how many eligible papers were identified. Typically, although several papers used similar outcome measures, they did not calculate and report on the relationship between FCR/FOP and engagement, making them not usable in this review.

In total, only two papers provided data relevant to answering the research question (see Table 3). Both papers reported the relationship between FCR and engagement, but no papers were identified that reported the relationship between FOP and engagement. Despite this, there were significant differences between these papers. Smith et al. [26] conducted a repeated-measures cross-sectional survey study with a population of female breast cancer survivors. The sample size was small, with only 30 participants completing the intervention, and the primary aim of the study was to explore the feasibility and uptake of a digital resource aimed at reducing FCR. There were several outcomes measured, but the ones of interest for this review were uptake and engagement of the digital resource and FCR. The study outlined its own criteria for calculating uptake and engagement and classified participants into usage groups. FCR was measured using the Fear of Cancer Recurrence Inventory Short-Form (FCRI-SF) [27]. This study aimed to identify any relevant correlates with engagement, which included calculating if there was any significant relationship between baseline FCR and engagement. Uptake was measured as the number of participants that agreed to take part in the study, and engagement was measured by grouping participants based on time spent using the resources, number of page views, and module/intervention completion. Results described a significant relationship between FCR and engagement, and participants were more likely to be grouped as low users if their baseline FCR was higher (OR = 1.26, 95% CI 1.004–1.585, *p* = 0.046). However, there was no reported calculation of any correlation between FCR and uptake of the resource.

Cillessen et al. [28] also classified users based on engagement with the resource and used the Fear of Cancer Recurrence Inventory [27] to measure FCR. Engagement was measured based on log data that reported time spent logged in and the number of assignments saved and submitted. This study also found that there was a significant relationship between usage and baseline FCR, with nonusers reporting increased baseline FCR (t 118 = 2.27, *p* = 0.03), and this was a medium to large effect (*D* = 0.69). FCR and adherence to the resource itself were also calculated but reported as non-significant, suggesting that FCR impacts the uptake/usage of a resource but not how well people adhere to the intervention once they have decided to use it.

### 3.2. What Is the Relationship Between FCR and Health Literacy and Digital Health Literacy?

**Figure 2:**
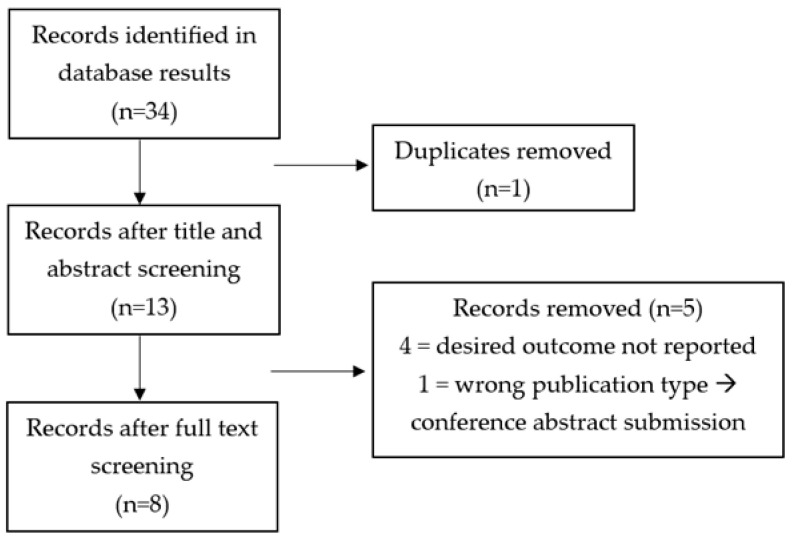
PRISMA flowchart for Objective 2.

The screening process for this second search is displayed in Figure 2. Similarly to the first question, although papers used similar outcome measures, often there was no specific relationship calculated and reported between the constructs of interest and, therefore, many studies were deemed not usable.

Despite this, a total of eight papers were identified as relevant (see Table 4). These studies reported the relationship between FCR/FOP and health literacy. No studies reported the relationship between FCR/FOP and digital health literacy. The study design was similar across these papers, with five out of the eight conducting a cross-sectional survey study [29,30,31,32,33] and one study reporting the results from a secondary analysis of data retrieved from a cross-sectional survey study [34]. Two studies [22,35] reported on a prospective cohort study.

Form NEO-FFI = NEO Five-Factor Inventory-3.

Sample size ranged quite significantly across all studies, from 155 participants in Tong et al.’s [31] study to 1749 participants in Zhang et al.’s [30] study. Participant eligibility also differed significantly between studies. There was a far greater number of female breast cancer patients recruited across the studies, but again, eligibility differed. For example, Halbach et al. [35] recruited newly diagnosed female breast cancer patients over the age of 65, Vandraas et al. [33] recruited female breast cancer survivors between the ages of 20 and 65, Tong et al. [31] recruited any female breast cancer patients over 18, Magnani et al. [34] focused on female cancer patients under age 55 that had been cancer-free for at least 5 years—although this study was open to any cancer type, over 80% of participants had been diagnosed with breast cancer, and Meng et al. [22] recruited both female and male participants, yet 63% of the sample were female and 53% were breast cancer patients. In contrast, Yang et al. [29] focused on male and female patients with advanced lung cancer. In this case, most participants (72.3%) were male. Clark et al. [32] recruited male and female head and neck cancer survivors, but 69% of participants were male. Zhang et al. [30] was the only study that recruited male or female cancer patients with any cancer type that had a more equal balance, with 54% being men. Additionally, Tong et al. [31] explored the influence of partner fears and, therefore, recruited only married women.

Further notable differences concern the outcomes measured across studies. Yang et al. [29], Zhang et al. [30], Tong et al. [31], Halbach et al. [35], and Meng et al. [22] focused on FOP rather than FCR and used the Fear of Progression Questionnaire—Short Form [36]. Clarke et al. [31], Vandraas et al. [32], and Magnani et al. [33] focused on FCR, but measures differed, with these studies using Fear of Relapse and Recurrence Scale [37], four items chosen from Concerns About Recurrence Scale [38], and a single-item screening question from the Fear of Cancer Recurrence Inventory [27], respectively.

All studies measured health literacy, and no studies were identified that focused on digital health literacy and FCR/FOP. However, once again, measures differed. Zhang et al. [30] and Tong et al. [31] both used the Health Literacy Management Scale [39]. The other studies used the Health Literacy Scale for Patients with Chronic Disease [29], Brief Health Literacy Screen [30], European Health Literacy Survey Questionnaire [35] and Single-Item Literacy Screener [34]. Vandraas et al. [33] used a 12-question version of the European Health Literacy Survey Questionnaire Short-Form, and Meng et al. [22] used a six-question version.

Despite these differences across studies, seven out of eight reported the same relationship: greater levels of FCR/FOP are associated with lower levels of health literacy. Statistical analyses differed across studies (see Table 4 for details). The only study with a different result was Zhang et al. [30]. In this study, the relationship was not significant (beta = −0.01, *p* = 0.699). It is relevant to consider that this was the largest study and the only study that recruited both male and female participants, had a similar percentage of both male and female participants (54% male), and did not specify a cancer type.

## 4. Discussion

FCR and FOP are significant emotional challenges for people who have received a cancer diagnosis and undergone treatment. Addressing the fear of the cancer coming back or progressing is also often described as an unmet need by cancer patients [17]. Therefore, it is important that we fully understand these constructs to improve patients’ experiences and quality of life. This scoping review aimed to explore these constructs in the context of digital resources by exploring two relevant questions: (1) what is the relationship between FCR/FOP and uptake and engagement with digital resources?; (2) what is the relationship between FCR/FOP and health literacy and digital health literacy?

A thorough database search was conducted to identify eligible studies to answer these questions. However, despite how widely studied the impact of cancer is, very few studies have been conducted in these areas. Only two studies reported on the relationship between FCR/FOP and uptake and engagement, yet an important concept in understanding FCR/FOP is avoidance coping. This is a commonly reported method of dealing with fears around cancer [40]. Evidently, digital resources are ineffective without initial uptake and continued engagement. Therefore, it seems increasingly relevant to explore the effect FCR/FOP has on engagement, yet this search revealed only two studies reported on this.

Further research appears increasingly relevant when considering that both Smith et al. [26] and Cillessen et al. [28] reported that higher baseline FCR is significantly associated with lower usage of digital resources. In this case, both resources being tested were aimed at reducing FCR and general patient distress. Therefore, this provides no insight into any other type of resource, for example, symptom trackers, information websites, forums, and peer support. As cancer care continuously moves to involve technology and digital resources, there is the potential that patients’ needs may not be addressed by the changing digital system if their fears prevent them from engaging with digital resources.

It should also be noted that both studies had relatively small sample sizes (N = 30 [26] and N = 125 [28]), and the participant population was not representative of all cancer types. Smith et al. [26] focused specifically on breast cancer, whereas Cillessen et al. [28] did not specify a cancer type for involvement, but 60.8% of participants had breast cancer. Therefore, it is not possible to ascertain the effect of FCR on engagement according to different cancer types. Again, as we move toward an increasingly digital age, we must conduct representative studies to explore and understand the impact of this construct on all cancer types. Furthermore, it is important to mention that both studies measured FCR and give no insight into the impact of FOP or any relationships. Therefore, this currently indicates a gap in our understanding.

Similarly, the search into the relationship between FCR/FOP and health literacy and digital health literacy highlighted a lack of research in this area. No studies focused on digital health literacy, and only eight studies were relevant to the research question. There was a more even mix between examining FCR and FOP among these studies, with five of the studies studying FOP and three studying FCR. Out of these studies, seven reported a significant relationship between FCR/FOP and health literacy. This indicated that there was a relationship between health literacy and fears around cancer, whether this was measured as FOP or FCR. Lower levels of health literacy were associated with increased levels of FCR/FOP. However, with such a limited number of relevant studies, it is not possible to draw firm conclusions from these results. Again, this highlights the need to fully understand how these constructs are related, what this means for understanding both FCR/FOP, and how to potentially improve patient outcomes.

The only study that did not report a significant relationship between health literacy and FOP was Zhang et al. [30]. This study had the largest sample size, with 1749 participants, and recruited patients with any cancer type over the age of 18 and male or female (54% male). The finding that there was no relationship between health literacy and FOP in this large study suggests that perhaps there are other factors influencing the results in other studies. One factor that appears to stand out among the other seven studies is that four of them specifically focus on women with breast cancer [31,33,35] or women with any cancer, but the majority being breast cancer [34], and they all reported a significant relationship. Due to the lack of research in this area, it is not possible to suggest that cancer type or sex mediate the relationship between FCR/FOP and health literacy, but the question does emerge when exploring existing research.

Despite the lack of studies that explored and reported the relationships between FCR/FOP and engagement and FCR/FOP and health literacy, studies were of a relatively high quality. Studies used reliable and validated outcome measures, eligibility and measure of exposure (in this case, cancer) were made clear, and analyses were appropriate for the research question of each study. Some of the main limitations were a lack of generalizability, the difficulty establishing causality in a correlational analysis, a lack of clear direction to limit or resolve these issues, and a small number of studies identified in the review. Therefore, the results discussed in this scoping review reflect quality research and make these results increasingly compelling, but do also suggest further research is required to improve generalizability and contribute further evidence to understand the nature of the relationship between FCR/FOP, engagement, and health literacy.

Arguably, the greatest takeaway from this scoping review is that more research is needed. Cancer rates continue to increase worldwide, with the expected global burden to increase by 27 million new cases per year by 2040 [41], and the cancer demographic is changing, with recent research showing a 79% increase in the global incidence of early-onset cancer [42]. Therefore, as cancer care adapts and patients are encouraged to take a greater role in their long-term care, digital resources are being introduced as a way of supporting patients [43]. If this is to be a successful endeavor, emotional experiences like FCR and FOP need to be taken into account in the design process and implementation of these digital resources. The studies discussed above display that this is a necessary relationship to explore, and yet little research has been done.

To design effective digital resources in the future, it may be crucial to first understand how baseline FCR and FOP can impact engagement. Additionally, it appears there may be a significant relationship between health literacy and FCR/FOP, with the greatest evidence so far for breast cancer patients. Not only does this suggest another risk factor for FCR/FOP, but the closely linked construct of digital health literacy may also be a concern. None of the studies identified focused on digital health literacy. Therefore, this remains an unanswered question. However, as digital health literacy combines both digital and health literacy, it is an even more complex and important construct when discussing the implementation of digital cancer resources going forward.

Aside from the requirement for further research to provide stronger evidence for the relationships described in this review, it also appears it may be important to explore these in specific patient groups. To design and implement effective resources and provide meaningful and effective support, we must understand the influence of other factors such as sex, age, and cancer type on the relationships between FCR/FOP and engagement and health literacy, as well as identifying any potential relationship with digital health literacy, as well.

## 5. Conclusions

This review explored two research questions: (1) what is the relationship between FCR/FOP and uptake and engagement with digital resources?; (2) what is the relationship between FCR/FOP and health literacy and digital health literacy? There appears to be some evidence to suggest that there is a significant relationship between FCR/FOP and these constructs. It appears that increased FCR may be related to lower engagement with digital resources. Furthermore, it seems that increased FCR/FOP may be associated with lower levels of health literacy. However, data are limited. It is not possible to draw firm conclusions on these relationships at this point. There was no data available to explore FOP and engagement or FCR/FOP and digital health literacy.

Our scoping review shows that there is a clear gap in research, and these findings suggest this may be an increasingly important area to explore. Future research must explore FCR and FOP in these contexts to identify factors that should be considered going forward in improving cancer care.

## Figures and Tables

**Table 1:** Search terms for Objective 1.

Operators	Terms
	Fear of recurrence OR fear of cancer recurrence OR fear of progression OR fear of cancer progression
AND	Cancer
AND	Engagement OR uptake OR barriers OR facilitators OR perception OR motivators
AND	Digital resources OR digital health OR virtual health care OR digital technology OR eHealth OR mHealth

**Table 2:** Search terms for Objective 2.

Operators	Terms
	Fear of recurrence OR fear of cancer recurrence OR fear of progression OR fear of cancer progression
AND	Cancer
AND	Health literacy OR digital health literacy or eHealth literacy

**Table 3:** Data extracted from search results Objective 1.

Author, Year, Country, Design	Participants	Study Aims	Outcomes Measured	Relevant Results	Quality Assessment
Smith et al., 2022 [26] Australia Repeated measures, survey data. Considered for prospective cohort study for this review.	N = 44 included in baseline calculations, N = 30 completed intervention. Women (mean age 55.3) with a breast cancer diagnosis, treated with curative intent, scored 13 or above on FCRI-SF.	Evaluate iConquerFear feasibility (uptake and engagement levels) and preliminary efficacy (FCR levels at 10 and 22 weeks) with breast cancer survivors.	Uptake = number that agreed to take part. Engagement = grouped based on time spent using resources, number of logins, page views, and module/intervention completion. FCR = FCRI-SF Anxiety = GAD-7 Intrusive thoughts = IES-R Negative metacognitions = MCQ-30 Depression = PHQ-9	Correlations with engagement = higher baseline FCR = increased likelihood of being classified as a low user (OR = 1.26, 95% CI 1.004–1.585, *p* = 0.046). No reported correlation between FCR and uptake.	10/10—relevant cohort study quality measures
Cillessen et al., 2020 [28] Netherlands RCT secondary analyses.	N = 125. Men and women (87.2% women), any cancer diagnosis (60.8% breast cancer), a score of 11 or above on the HADS scale (mean score 17).	Examine the usage of a digital mindfulness-based cognitive therapy resource in relation to outcome à explore baseline characteristics as predictors of uptake and adherence. Explore adherence related to treatment outcome.	Log data = grouped into usage levels by time at login and out, number of assignments saved and submitted, and emails. Sociodemographic characteristics: age, education, cancer type, treatment intent. Psychological predictors: Psychological distress = HADS Positive mental health = MHC-SF Rumination = RRQ FCR = FCRI Mindfulness skills = FFMQ-SF Personality = NEO-FFI	Nonusers had higher levels of baseline FCR compared with users (t 118 = 2.27, *p* = 0.03). Medium to large effect (*D* = 0.69). No other differences between users and nonusers at baseline. No significant relationship between FCR and adherence among users.	10/13—relevant RCT quality measures

Abbreviations: FCRI-SF = Fear of Cancer Recurrence Inventory—Short Form GAD-7 = General Anxiety Disorder-7 IES-R = Impact of Event Scale—Revised MCQ-30 = Metacognitions questionnaire-30 PHQ-9 = Depression module of Patient Health Questionnaire HADS = Hospital Anxiety and Depression Scale MHC-SF = Mental Health Continuum—Short Form RRQ = Rumination-Reflection Questionnaire FFMQ-SF = Five Facet Mindfulness Questionnaire—Short The other eligible study was conducted by Cillessen et al. [28]. This was a larger study with a total of 125 participants, and the eligibility criteria were much less specific, with male and female cancer patients diagnosed with any type of cancer being eligible. Despite this, there were more female patients with breast cancer in this study than males or other cancer types. This paper also had a different aim, which was to identify the relationship between baseline characteristics and usage and adherence to an intervention, in this case, a digital resource aimed at reducing overall distress in cancer patients. This study conducted a secondary analysis of data retrieved by an RCT, exploring the effectiveness of the intervention.

**Table 4:** Data extracted from search results Objective 2.

Author, Year, Country, Design	Participants	Study Aims	Outcomes Measured	Relevant Results	Quality Assessment
Yang et al., 2023 [29] China Cross-sectional, surveys	N = 230, N = 220 completed all questionnaires. Men or women (72.3% men), diagnosed with primary lung cancer, non-small cell lung cancer at clinical stage IIIb-IV, or small cell lung at the extensive stage, over 18 (mean age 53.75), aware of disease, writing and reading abilities.	Describe FOP among advanced lung cancer patients and explore relationships among family support, health literacy, and FOP.	FOP = FOP-Q-SF Symptom experience = MDASI-LC Family support = FSQ Health literacy = Health Literacy Scale for Patients with Chronic Disease Sociodemographic and clinical variables = age, gender, education, monthly household income, marital status, health insurance, time since diagnosis, treatment modalities, history of surgery, history of cancer progression	Higher health literacy was correlated with lower FOP (beta = −0.337, *p* = 0.002). Higher health literacy was correlated directly with lower FOP through better symptom experience (beta = −0.121, *p* = 0.009). The model accounted for 37.0% of the variance among FOP.	7/8—relevant cross-sectional quality measures
Zhang et al., 2023 [30] China Cross-sectional, surveys	N = 1749. Men and women (54% male) over 18 (18% aged 18–45, 44.6% aged 45–60, 37.5% aged 60+), diagnosis of cancer, without cognitive impairment or mental disorder.	Construct a structural equation model to explore health-related quality of life, health literacy, social support, self-efficacy, and fear of progression.	General information = demographics, blood type, occupation, monthly income, medical burden, place of residence, religious beliefs, main caregivers, family companionship, mood state, efforts to treat illness, and decision-maker for treatment plans. Health literacy = HeLMS Health-related quality of life = EORTC QLQ-C30 FOP = FoP-Q-SF Social support = SSRS Self-efficacy = SUPPH	In the structural equation model, the path between health literacy and FOP was not significant (beta = −0.01, *p* = 0.699).	8/8—relevant cross-sectional quality measures
Tong et al., 2024 [31] China Cross-sectional, surveys	N = 155. Women diagnosed with breast cancer, mental awareness, good reading, and communication skills in Chinese, over 18 (mean age 53.92), married.	Investigate the levels of FCR in breast cancer patients and partners and explore the correlation with the FCR of the spouse, family resilience, and cancer health literacy.	Sociodemographic characteristics = age, marital status, educational level, surgical procedure, body mass index, payment methods for medical expenses, disease stage FOP = FoP-Q-SF FOP partners = FoP-Q-SF/P Family resilience = FaRE Health literacy = HeLMS	FCR negatively correlated with health literacy (*r*(153) = −0.538, *p* = 0.01). In multiple linear regression, health literacy is a significant predictor of FCR. (beta = −0.1, *p* = 0.029).	7/8—relevant cross-sectional quality measures
Clarke et al., 2021 [32] Ireland Cross-sectional, surveys	N = 395. All head and neck cancer survivors eligible, aware they had cancer, not receiving palliative care, had not developed a second invasive cancer, had completed primary treatment for HNC, not receiving treatment for recurrence or secondary cancer, considered cancer-free for at least 4 months prior, no medical reason it would be inappropriate to contact. Men (69%), 33% aged 50–59, 29% aged 60–69.	Investigate the sociodemographic and clinical profile of health literacy and associations between health literacy and health-related quality of life, self-management behaviors, and FCR in a population-based sample of HNC survivors.	Sociodemographic data = highest level of education, relationship and employment status, residential status, residential location, medical card status, current smoking status, alcohol use (AUDITC), and comorbidities. NCRI provided sex, age, cancer site, treatments received, and stage of disease. Health literacy = Brief Health Literacy Screen Health-related quality of life = FACT-G and FACT-HN Self-management = HEIQ FCR = FRRS	Unadjusted model FCR did not differ between those with adequate and inadequate health literacy (adequate M: 13.33: 95% CI 12.70 to 13.97; inadequate M: 14.20: 95% CI 13.50 to 14.90; *p* = 0.071). Adjusted model FCR was significantly higher in those with inadequate health literacy (Coef 0.98; 95% CI 0.04 to 1.92, *p* = 0.040).	8/8—relevant cross-sectional quality measures
Vandraas et al., 2022 [33] Norway. Cross-sectional, surveys	N = 1355. Female survivors of breast cancer aged 20–65 years, when diagnosed with breast cancer in 2011–2012 (mean age at survey 59.9 years), had to be free of prior or successive malignant disease. Average 8 years since diagnosis.	Describe health literacy in a large cohort of long-term survivors of breast cancer and explore factors associated with health literacy.	Health literacy = HLS-Q12 Socioeconomic data = education, financial income year prior to the survey, living arrangements, employment Somatic comorbidity = Charlson comorbidity index Personality = Eysenck Personality Questionnaire short version Cancer-related data = age at diagnosis, pathological state, hormone receptor, HER-2 status, information on surgical treatment. Pain intensity and cognitive function = EORTC-QLQ-C30 version 3 Neuropathy = SCIN Arm and breast symptoms = EORTC QLQ-BR23 Fatigue = Chalder’s Fatigue Questionnaire Sleep problems = two items from Nord-Trondelag Health Study Depressive symptoms = PHQ-9 Anxiety symptoms = GAD-7 FCR = four items from CARQ	FCR is inversely associated with health literacy (*B* = −0.15, *p* = <0.01). In multivariate analysis, FCR is still inversely associated with health literacy (*B* = −0.08, *p* = <0.01).	8/8—relevant cross-sectional quality measures
Magnani et al., 2022 [34] France Secondary analyses of cross-sectional study, surveys	N = 1153. Women diagnosed with non-metastatic good-prognosis cancer (81.8% breast cancer), aged 55 or less at diagnosis (mean age 44 years), with no recurrence or progression in 5 years following diagnosis.	Document the prevalence of self-reported FCR and associated factors in younger women with no recurrence. Study focused on sociodemographic characteristics, cancer-related sequelae, psychosocial consequences, and survivorship care—highlighting the role of GP.	FCR = single-item screening question from the Fear of Cancer Recurrence Inventory Sociodemographic characteristics = age at diagnosis, level of education, employment status, perceived financial precariousness Health literacy = SILS GP follow-up care = ask participants about contact. Cancer-related symptoms = ask participants whether they had been informed Cancer-related sequelae = ask participants this as a single question Body image = four items Body Image Scale Sexuality = one question Relation and Sexuality Scale Cancer-related fatigue = EORTC QLQ Quality of life = SF-12 Anxiety and depression = HADS	Mild FCR associated with a higher likelihood of reporting limited health literacy. OR = 1.81, 95% CI 1.34–2.44, *p* = <0.001.	7/8—relevant cross-sectional quality measures
Halbach et al., 2016 [35] Germany Prospective cohort study.	N = 1359 at baseline, N = 445 at first follow-up, N = 344 at second follow-up. Breast cancer patients are eligible if their inpatient surgery for newly diagnosed breast cancer occurred between 1 February and 31 August 2013, with at least one malignancy and at least one postoperative histological evaluation. Current analyses focused on women over the age of 65.	Investigate the distribution of health literacy levels throughout breast cancer treatment in elderly women diagnosed with breast cancer, investigate FOP levels throughout treatment, and analyze the association of health literacy with FOP throughout treatment.	Health literacy = HLS-EU-Q16 FOP = FoP-Q-SF Sociodemographic data = age, children, educational level, number of comorbidities, live with partner. Clinical data = tumor size, lymph nodes, and metastases added by clinical personnel Psychosocial data = MOS-SS	Inadequate and problematic health literacy was significantly associated with higher levels of FOP, with 6.50 points (*p* = 0.000) and 3.02 points (*p* = 0.001). Appeared to be no change in relationship across time follow-up points.	7/9—relevant cohort quality measures
Meng et al., 2021 [22] Germany Prospective cohort study.	N = 449 at baseline, N = 418 at follow-up 1, N = 401 at follow-up 2. Complete data available for N = 395. Rehabilitation patients with breast, prostate, or colorectal cancer, over 18 years old, sufficient knowledge of German, and no severe uncorrected visual impairment. 53% with breast cancer, 63% female.	Explore the presence of health literacy in oncological rehabilitants, investigate correlations between HL and sociodemographic and clinical parameters of patients, and provide an insight into the correlations between HL, psychological stress, physical functioning, global quality of life, subjective ability to work, and employment prognosis.	Health literacy = 6-item short-form HLS-EU-Q6 of HLSEU-Q Fear of progression = FOP-Q-SF Physical functioning, global quality of life = EORTC-QLQ-C30 Psychosocial support needs = a single-item dichotomous response format used to ask whether there was a current need for support Subjective ability to work and employment prognosis = WAI	Higher HL is associated with lower progression anxiety (β = −0.33, *p* < 0.001).	7/8—relevant cohort quality measures

Abbreviations: FOP-Q-SF = Fear of Progression Questionnaire—Short Form MDASI-LC = MD Anderson Symptom Inventory FSAQ = Four Systems Anxiety Questionnaire HeLMS = Health Literacy Management Scale EORTC QLQ-C30 = European Organization for Research and Treatment of Cancer Core Quality of Life Questionnaire SSRS = Social Support Rating Scale SUPPH = Strategies Used by People to Promote Health FaRE = Family Resilience Questionnaire FACT-G = Functional Assessment of Cancer Therapy FACT-HN = Functional Assessment of Cancer Therapy Head and Neck. HEIQ = Health Education Impact Questionnaire FRRS = Fear of Relapse and Recurrence Scale. HLS-EU-Q16 = European Health Literacy Survey Questionnaire. MOS-SS = Medical Outcomes Study Social Support Survey. HLS-Q12 = European Health Literacy Survey Questionnaire Short-Form SCIN = Scale for Chemotherapy Induced Neurotoxicity EORTC-QLQ-BR23 = European Organization for Research and Treatment of Cancer Core Quality of Life Questionnaire Breast Cancer PHQ-9 = Depression module of Patient Health Questionnaire GAD-7 = General Anxiety Disorder-7 CAR = Concerns About Recurrence Scale SILS = Single-Item Literacy Screener SF-12 = 12-Item Short-Form Survey HADS = Hospital Anxiety and Depression Scale. WAI = Work Ability Index. HLS-EU-Q6 = 6-item short-form of European Health Literacy Survey Questionnaire.

## Data Availability

No new data were created or analyzed in this study. Data sharing is not applicable to this article.
